# Experiences of an Autism Diagnosis in Adulthood: The Role of Grassroots Epistemology in Clinical Settings

**DOI:** 10.3390/jcm14124315

**Published:** 2025-06-17

**Authors:** Weronika Superson, Anna Prokopiak, Maciej Wodziński

**Affiliations:** 1 Department of Special Psychopedagogy, Institute of Pedagogy, Faculty of Pedagogy and Psychology, Maria Curie-Skłodowska University, Gł˛eboka 43, 20-612 Lublin, Poland; wersuperson@gmail.com (W.S.); anna.prokopiak@mail.umcs.pl (A.P.); 2 Department of Ontology and Epistemology, Institute of Philosophy, Faculty of Philosophy and Sociology, Maria Curie-Skłodowska University, Maria Curie-Skłodowska Sq. 4, 20-031 Lublin, Poland

**Keywords:** autism spectrum, adulthood, diagnosis, first-person perspective, relationships, grassroots epistemology, community knowledge, clinical practice

## Abstract

**Background:** Our research problem mainly concerns the following question: what are the consequences of an autism spectrum condition diagnosis for everyday functioning and self-understanding? **Method:** The research methodology is based on a semi-structured interview, which allows respondents to share their experiences freely while maintaining the structure and standards necessary to collect consistent data. The research was conducted remotely, using the ZOOM platform and the Messenger application. Six individuals with autism spectrum condition (ASC) diagnosed in adulthood, selected intentionally, participated in the study. Interviews lasted between 20 and 60 min. **Results:** The analysis of the experiences of individuals with ASC diagnosed in adulthood highlights the complexity of the diagnosis process and its far-reaching consequences. The diagnostic process, which varied in time and depended on the availability of specialists, was often evaluated positively. Obtaining an ASC diagnosis proved to be a crucial moment for the interviewees, enabling them to better understand themselves and their needs. It led to a better adjustment of their work, education, and private life environment. Relationships with loved ones tended to remain unchanged or improved, highlighting the importance of the diagnosis in improving the understanding and acceptance of individuals with ASC. **Conclusions:** The study sheds light on the positive impact of diagnosis on self-awareness and quality of life for individuals with ASC, revealing the need to improve the availability of specialised diagnostic and support services. Further research should focus on the development and adaptation of support methods to meet the individual needs of individuals with ASC and on promoting awareness of ASC in the community and among mental health professionals.

## 1. Introduction

Autism Spectrum Condition (ASC) refers to a range of neurodevelopmental differences characterised by atypical social communication, restricted interests, and sensory sensitivities [1]. Both main diagnostic manuals, the International Classification of Diseases (ICD) [2] and the Diagnostic and Statistical Manual of Mental Disorders (DSM) [3], define autism spectrum as a disorder and through persistent social communication difficulties and repetitive behaviours but differ in classification. The DSM-5 uses a three-level severity scale based on support needs, while the ICD-11 focuses on intellectual and language impairments without rating severity. The DSM-5 offers detailed, research-oriented criteria; the ICD-11 opts for flexible language suited to diverse clinical contexts. While both acknowledge the spectrum’s heterogeneity, the DSM-5 prioritises standardisation, and the ICD-11 emphasises broad clinical usability.

However, in this article, we adopt the term condition rather than disorder to reflect a neurodiversity-informed perspective. We understand neurodiversity as a conceptual framework that views neurological differences—such as autism—as natural variations of human cognition, rather than pathologies. Rooted in the social model of disability, it supports a non-deficit view of autism and informs our commitment to valuing divergent ways of being and knowing. Therefore, this approach seeks to describe autism as a variation of human functioning rather than a pathological deficit [4]. We are aware that the use of the term condition instead of disorder is not consistent with the current definitions used in the DSM or ICD classifications and that not the entire autism community identifies with this approach. We acknowledge the ongoing tensions between clinical models, self-identification, and caregiver perspectives in this area. These groups often represent different viewpoints based on the need to place different needs at the centre (e.g., the need for a medical diagnosis, the need to define one’s identity, or the need for state support).

The use of the term condition is a deliberate decision on our part, motivated by a desire to move away from the deficit-based perspective on the autism spectrum that currently tends to dominate media, social, and academic messages.

The experience of disability for adults on the autism spectrum is a scarcely researched topic. Many people perceive individuals with ASC as dependent on other people, whereas, in fact, a large proportion of them function completely independently. They are independent, intelligent, and, above all, self-aware individuals. Furthermore, research studies conducted by Anderson-Chavarria [5] and Ripamonti [6] challenge the perception of autism as a disability, instead highlighting the unique cognitive strengths of people with autism. Especially from an evolutionary psychology perspective, autism, understood as a specific, often hyper-systemising cognitive style, is sometimes seen as a cognitive and functional advantage rather than a disadvantage [7]. However, it is important to remember that interpreting autism as a functional advantage is a contested view within both clinical and neurodiversity communities. While highlighting strengths such as better pattern recognition, memory specificity, or systemising abilities may help challenge deficit-based models, this framing can hide the reality of co-occurring conditions (e.g., anxiety, sensory processing difficulties, or executive functioning challenges) that may significantly impact quality of life. Many autistic individuals require tailored support across education, employment, and healthcare settings—needs that can be overlooked if autism is portrayed in overly idealised terms. Yet, Simon Baron-Cohen [8] in his work also emphasises that referring to people on the spectrum as disabled is often associated with negative values and instead advocates using terms that refer to differences rather than deficits [9,10,11].

This study focuses on adults diagnosed with ASC later in life—often after establishing careers and relationships—due to both empirical and conceptual reasons. As previous research shows [9,11], this group remains underrepresented, despite a growing number of adult diagnoses. Their retrospective accounts offer unique insight into identity reconstruction and the reinterpretation of life experiences. These narratives are especially valuable for understanding how diagnosis affects autonomy, self-understanding, and social integration—core themes of this study.

In this article, we analyse the results of our own qualitative research study aimed at exploring the experience of disability by individuals on the autism spectrum diagnosed in adulthood. This includes an analysis of the subjects’ lives before receiving the diagnosis, the diagnostic process, the first impressions and experiences following the diagnosis, the subjects’ lives after the diagnosis, and an evaluation of the effects of the diagnosis.

Katarzyna Stein-Szała’s analysis of the reports found that individuals with ASC often experience loneliness and social isolation but crave friends and close relationships. It was also found that only a minority of them have spouses. Individuals with ASC, despite having many interests, spend most of their time at home, with most stating that they would like to spend this time with other people [12]. These findings underscore the discrepancy between common perceptions of autistic adults as socially uninterested and their actual desire for meaningful social connection. Recognising this gap highlights the importance of adopting a grassroots epistemology, which centres on the lived experiences and interpretations of autistic individuals themselves rather than relying solely on externally imposed diagnostic frameworks. Such first-person perspectives enable a more nuanced analysis of social withdrawal—not as an inherent trait of autism but as a dynamic response to past relational experiences, environmental barriers, and unmet support needs. As autistic self-advocate Jim Sinclair succinctly observed, many autistic people spend their lives “reaching into [the neurotypical] world and making contact” despite being persistently misunderstood as incapable of doing so [13].

This study focuses on analysing the challenges of diagnosis and the transition of individuals with ASC from childhood to adulthood, taking into account gender differences and the camouflage effect. The term camouflage or camouflaging refers to conscious or unconscious strategies used by autistic individuals to mask or compensate for their traits in order to fit social expectations [14,15]. While camouflaging may support short-term social adaptation, it is also associated with increased stress, delayed diagnosis—especially in women and gender-diverse individuals—and long-term psychological costs [16,17]. Among people with ASC, camouflaging their condition has become a popular phenomenon. Fearing social misunderstanding of their specific functioning, individuals on the spectrum hide their atypical behaviour and the very fact of having a diagnosis. Under such conditions, defining their individual and social identity becomes even more difficult for them. People who camouflage themselves often emphasise that they are accompanied by a sense of “living a lie” and “denying themselves”, resulting from the need to conform to social demands and expectations and a reluctance to stand out from others.

Importantly, research into the experiences of individuals with ASC in adulthood points to significant limitations in the available data due to difficulties in recruiting a broad and representative sample of participants [18], often resulting in a focus on online research with the participation of people without intellectual difficulties [19].

In addition, there is a notable gender imbalance among study participants. Although both women and men with ASC experience similar difficulties in communication and social behaviour, differences in the manifestation of stereotypical behaviour and additional psychological problems, such as depression and anxiety disorders, are more common in women prior to diagnosis [20]. Women with ASC are more likely than men to attempt to hide their difficulties, which may manifest as better social and language skills and fewer hyperactive behaviours.

All of this points to possible shortcomings in current diagnostic tools that may not fully account for the specific characteristics of ASC in women [21]. This results in a delay in getting a diagnosis and receiving appropriate specialist help [1,16,22]. It is also important to highlight the difficulties in accessing diagnoses and appropriate support for adults with ASC, particularly women, for whom the diagnostic process can be particularly complex [23].

Further research is needed into the experiences of individuals with ASC in the context of gender differences, as well as into the importance of including a neurodiversity perspective when approaching ASC as one of the pathways of human development. The impact of diagnosis on understanding one’s difficulties, accessing support, and building social relationships has not been sufficiently explored to date.

Knowledge about autism is developing dynamically. Through ideas such as neurodiversity or an appreciation of the role that experiential and lived, first-person experience plays in psychiatry, further perspectives are being incorporated into autism knowledge, organised hierarchically in a top-down model, to help understand other aspects related to diagnosis, therapy, and the subsequent lives of people on the spectrum. Knowledge produced in a “grassroot epistemology” model, i.e., taking into account and prioritising the views of autistic people and their close ones, ensures that this knowledge is not a stagnant monolith. While new theories and discoveries in the disciplines of the specific sciences are constantly emerging, it is now no longer possible to ignore the experiences of specific individuals who live and experience their diagnosis in different ways. This study is grounded in the idea of grassroots epistemology, which prioritises knowledge emerging from the lived experiences of marginalised individuals rather than from top-down clinical or institutional authority [24,25]. Changing the epistemological position is, in our opinion, a key aspect in the field of autism research today. The current power/knowledge relations, and consequently the social and institutional structures that determine what kind of knowledge about autism and people on the spectrum is produced, are very often the result of relationships, frictions, and tensions between multiple actors in the knowledge-generating process (political institutions, thinktanks, research funding entities, private actors, and autism-focused communities themselves). For decades, these balances of power have shaped the contemporary epistemic field, and only recently has the academy begun to appreciate the contributions of individuals on the spectrum themselves and their families to the knowledge creation process. The voices of people on the spectrum are slowly beginning to be heard more and more, and researchers are beginning to recognise that so-called experts-by-experience can make important contributions to knowledge production, including at the clinical level. Within autism research, this approach challenges traditional deficit-based narratives and centres the voices of autistic people as active contributors to the understanding of their own condition. Therefore, the concept of grassroots epistemology refers to a bottom-up, inclusive model of knowledge creation that prioritises the lived experiences, collective interactions, and everyday practices of communities. In contrast to conventional epistemological models that often favour expert-driven or institutional knowledge, this approach recognises the intellectual contributions and authority of marginalised groups and activists, particularly as they respond to pressing social, environmental, or political issues.

Our study demonstrates that bridging the gap between contrasting knowledge paradigms requires embracing a grassroots approach—one that centres on the interpretive practices and everyday meaning-making of autistic individuals and their families.

A particular type of exclusion affects women. Single women with autism spectrum disorders often face misunderstanding and are seen as eccentric or suspicious. Unlike single men, they are required to be sociable. Problems start as early as school, when peers ridicule their unusual appearance, upbringing, or specific behaviour. These negative experiences have long-lasting effects in adulthood, even though individuals with ASC gradually learn to establish an understanding with others and recognise their positive qualities. They have difficulties in maintaining healthy relationships with people their own age and find it easier to relate to older people when they are young due to their more developed intellectual maturity. However, as they grow up, they feel more comfortable around younger people because they do not reach emotional maturity as quickly. As a result, they find it difficult to find people who are both open and have a youthful approach to life. It is important to understand that they need support and understanding in establishing and maintaining social relationships but also physical and emotional space for rest and solitude when they need it. Mutual support and acceptance can help individuals with ASC participate more fully in society and build fulfilling relationships with others [26,27].

Although the theme of social misunderstanding and the exclusion of single women with ASC does not constitute a separate analytical category directly derived from the empirical material, it has been included as a contextual background for interpreting certain individual narratives. The relational status of participants—whether in a partnership or single—may influence the timing of the decision to seek a diagnosis. While this variable was not systematically coded, its significance emerges in participants’ accounts concerning their motivations for diagnosis and their self-understanding following its confirmation.

Research into self-awareness among individuals with ASC reveals a complicated relationship between how they see themselves and how they connect with others. Some studies highlight that many of them possess a deep understanding of their emotions and sensations [28,29] while others show that they struggle to understand their identity within social situations [30,31,32]. This tension often arises from a mismatch between their internal experiences and what society expects from them [33]. Navigating the diagnostic process is then highly influenced by the interpretation of one’s condition and building meaningful social relationships.

The literature highlights three central challenges: (1) delayed diagnosis and its gendered dimensions, often intensified by camouflaging strategies; (2) discrepancies between public perceptions and lived experiences of social situations; and (3) the role of self-awareness in psychosocial functioning. In our opinion, addressing these requires a shift toward a grassroots epistemology that centres on the first-person narratives and everyday experiences of autistic adults. By focusing on subjective accounts, this study aims to expand current clinical and social understandings of autism through the lens of lived experience.

## 2. Methods

The research problem boils down to the following question: What are the consequences of receiving an ASC diagnosis in adulthood for individuals’ everyday functioning and self-understanding?

In order to answer the question posed in this way, we decided to conduct a series of interviews with individuals on the autism spectrum, recognising that their first-person accounts and lived experiences would bring the most relevant content to analyse the problem. The research question serves as the starting point for a broader inquiry into how a diagnosis affects identity, social relationships, and the adaptation of one’s environment to individual needs. The aim of the study is not merely to describe diagnostic events but to conduct an in-depth analysis of the meanings that participants assign to their experience of diagnosis. In this context, the use of a qualitative approach based on semi-structured interviews and thematic analysis enables the exploration of subjective perspectives and treats diagnosis as a biographical event with both epistemic and existential dimensions.

Within the adopted methodology, expertise based on personal experience becomes crucial as it allows for a fuller understanding of the complexity of the situation and experience of people with ASC, which play an important role in both the clinical diagnosis and subsequent management process. This method allows unique perspectives to be highlighted that may be overlooked in traditional research approaches, consequently contributing to the development of more effective personalised clinical practices and improving the quality of life of autistic people. As argued in the qualitative research literature, first-person narratives offer not only access to subjective meaning-making but also challenge dominant knowledge hierarchies by valuing experiential knowledge as a legitimate and necessary epistemic source [34,35].

Giving a voice to people on the spectrum themselves is an important factor in closing the current research gap consisting in the fact that the conducted research is guided by priorities that do not reflect the real expectations of the community and treats individuals on the spectrum as “research objects” instead of full-fledged subjects of social and research life. In this study, the inclusion of autistic voices was implemented through semi-structured in-depth interviews, in which participants were encouraged to reflect freely on their experiences of diagnosis and its personal and social implications. Participants were also invited to provide feedback on the preliminary thematic interpretations, ensuring that the findings remained grounded in their own perspectives and meanings. This participatory orientation not only recognises autistic individuals as knowledge contributors but also aligns with ethical research practices grounded in respect, reciprocity, and epistemic justice. In this study, semi-structured interviews were conducted using a guide composed of open-ended and semi-open questions structured around key phases of the ASC diagnostic experience: the pre-diagnostic period, the moment of diagnosis, emotional reactions, post-diagnostic changes in everyday life, and processes of self-understanding and identity redefinition. The interview protocol was designed to provide a thematic framework while allowing flexibility to explore topics that participants found personally significant. This approach enabled the identification of shared patterns across narratives while also preserving the uniqueness of individual accounts. Although each interview followed a distinct trajectory, all included core initiating questions regarding the motivations for seeking a diagnosis, the course of the diagnostic process, the social reception of the diagnosis, and the impact of the diagnosis on participants’ functioning [36].

First-person perspectives were central not only to data collection but also to analysis. Participants’ narratives were treated as meaningful in themselves, allowing themes to emerge inductively from their lived experiences. This approach made it possible to capture nuanced dimensions of diagnosis and identity formation that might be missed in clinician-driven frameworks. Interviews were conducted remotely, mainly via the ZOOM platform and on one occasion using the Messenger application. Residing in their own homes, the interviewees connected with the researcher using the aforementioned software solutions, and the interviews took place over a four-month period. At the beginning of each interview, the subjects gave their consent to participate in the study and to be recorded. Respondents were informed that they would remain anonymous. They were also informed of their rights to withdraw from participation in the study or the possibility of modifying and completely withdrawing the interview data, in accordance with applicable data protection laws. Due to the non-invasive and non-experimental nature of the study, formal approval from the university’s research ethics committee was not required. Nevertheless, if the necessity arose, participants in the study were also able to receive professional support from the university and its experts. They showed great interest in the purpose of the research. They inquired about the possibility of reading the study report at a later date. The decision to use remote interviews was not only based on logistical convenience but also aimed to create a familiar and safe environment for participants, which is particularly important when exploring sensitive, identity-related topics with individuals on the autism spectrum.

The study was conducted with 6 intentionally selected individuals on the autism spectrum who had received a diagnosis in adulthood. Most subjects had a very high level of communication skills, which allowed for fully engaged interviews. These lasted between 20 and 60 min. The content of the interviews was transcribed, followed by a qualitative analysis, leading to the emergence of several common, recurring themes and categories, which we discuss in the Results section. Table 1 presents descriptions of the interviewees: their age, gender, education, marital status, place of residence, place of work, and the number of children they had at the time of the survey and the receipt of the diagnosis.

We employed a purposive sampling strategy, based on the assumption that the individuals best positioned to provide in-depth insights are those who have themselves experienced a late diagnosis of ASC. Inclusion criteria were as follows: (1) a confirmed diagnosis of autism spectrum condition received after the age of 18; (2) the ability to participate in the interview without the presence of a caregiver or assistant; and (3) sufficient verbal communication skills to freely share one’s experience. Exclusion criteria included (1) a prior diagnosis of ASC in childhood; (2) a co-occurring intellectual disability that precluded participation in a direct interview; and (3) a lack of consent to participate in the study or to the recording of the interview.

The table presenting basic sociodemographic characteristics of the participants was included not only to provide a transparent overview of the sample but also to serve as a contextual background for the interpretation of the selected interview excerpts. Variables such as educational attainment or life circumstances (e.g., being in a relationship versus being single) proved relevant in the analysis of certain narratives, particularly in relation to access to diagnosis, the availability of social support, and adaptive strategies.

Of the six participants included in the study, five had attained higher education qualifications, while one had completed secondary education. All individuals function within the range of normative intellectual ability. No comorbid clinical diagnoses were identified among the participants beyond the diagnosis of autism spectrum condition (ASC).

## 3. Results

The results of the study were derived through thematic analysis, conducted using an inductive approach [37]. The analytical process involved repeated readings of the transcripts, open coding, and the grouping of codes into categories that reflected recurring patterns in participants’ narratives. This section presents the key areas of meaning that emerged from the analysis, illustrated with direct quotations from the interviews. These categories were not predefined but developed through an interpretive engagement with the empirical material and they correspond to the main dimensions of the experience of receiving an ASC diagnosis in adulthood.

### 3.1. The Need for Social Participation

Our study revealed a complex relationship between educational level and motivation for social inclusion. The term “motivation” refers to the subjectively indicated reasons and initiating factors that led individuals to seek a diagnosis—both in intrapsychic terms (e.g., the need to make sense of one’s identity) and in social terms (e.g., external pressure, difficulties in the workplace, or interpersonal relationships). The analysis indicated that higher education and awareness of the benefits of acquired skills fostered a greater interest in social participation.

The communication and social difficulties described by participants included both an inability to read nonverbal cues and others’ intentions as well as a pervasive sense of alienation and a lack of belonging in social interactions. While these experiences were diverse in form, they shared a common dimension of disorientation and the constant need to monitor one’s own behaviour. Individuals with ASC, despite experiencing difficulties in collegial relationships, expressed the need to maintain social contacts, seeking membership in social groups with similar experiences. No differences were observed in the types of online activities according to educational level. The interviewees with ASC, identifying themselves with their environment, showed a tendency to be open to the environment and to actively participate in it. The need to establish relationships and seek a life partner, both online and offline, was also reported. The risk of over-disclosing personal content online and difficulties in initiating and maintaining social contacts due to communication and social deficits were noted. Individuals with ASC received limited feedback in interactions, which could hinder relationship building. A recurring theme in their narratives was the pursuit of happiness, often framed in terms of personal satisfaction and the quality of social functioning. Participants frequently emphasised values such as independence, self-reliance, family, and a sense of security. Those who expressed a strong desire for intimate relationships tended to highlight values linked to fulfilling social connections. In contrast, values related to pleasure and intense emotional experiences were mentioned less often or with ambivalence, which may reflect a tendency to avoid emotional discomfort and to prioritise stability and introspection. Despite challenges in establishing social connections, several participants described online activity as a meaningful way of meeting social and emotional needs [37].

### 3.2. Experiences Before the Diagnosis Process

Upon the analysis of the experiences of individuals with ASC related to the process of diagnosis, it can be observed that the majority of respondents sought psychological support before officially being diagnosed with the disorder. Professionals, as in the case of Milena, were often the first ones to draw attention to behaviours suggestive of ASC, particularly in the context of transgender people. Adam, experiencing a mental health crisis, identified himself with the autobiography of a person with ASC, which led him to seek specialist support:

“Let’s just say I fell into a sort of mental crisis, or a psychological crisis. I don’t know how to define it. Well, for the first time in my life I started to seek psychological help. At first, I had the impression it didn’t bring any results. Then, for some reason, I started to take an interest in the topic of the spectrum and autism, and I read a book. I was very impressed with the book, because I had the feeling that 90 per cent of it was the story of a man who was diagnosed there at the age of 40 and described his life. And what he was describing there seemed very close to me. So I just went to this local mental health clinic, because I was also having this kind of crisis, but I didn’t get any specifics, it wasn’t satisfying to me and I decided that I would verify it somewhere else and I went to this kind of centre that specialises in autism and I made a diagnosis there and it was confirmed by specialists there.”

Adam’s testimony is evocative and relevant. His narrative illustrates a broader theme observed in several interviews: the role of diagnosis as a form of emotional regulation and narrative coherence. For Adam, the process of identifying with another autistic person’s life story served as a powerful moment of recognition and validation.

Both Marta and Ania, despite years of therapy, were only diagnosed in adulthood, after becoming interested in ASC themselves. Marta mentioned the stereotypical perception of autism, which delayed her decision to be diagnosed:

“I generally had this idea that I was autistic somehow in high school, but my stereotype of autism that I had in my head was the kind of autism that is typically seen in boys and one with, let’s say, a lot of difficulty in relationships. I thought maybe I had some of that, but not really.”

Marta’s comments on stereotypes are valuable, as they highlight the impact of gendered expectations and normative models of autism on the diagnostic process. Her hesitation was caused by the belief that autism is mainly characterised by visible social difficulties and stereotypically “male” traits. This reflects a broader issue in autism diagnosis—the underrecognition of atypical presentations, particularly among women, who often engage in camouflaging strategies that mask outward signs of autistic traits. Often the diagnosis was preceded by a lengthy search for a suitable venue and specialist, illustrating difficult access to diagnostic services for adults. The diagnostic process took different lengths of time, ranging from a few weeks to several months, which depended on the availability of specialists and diagnostic procedures. Most study participants assessed the diagnostic process positively, highlighting the professionalism and friendly atmosphere, but they often lacked a thorough discussion of test results. In the case of Daria, her experience was mixed, due to the unprofessional comments she received. The diagnostic barriers described by participants exhibited both commonalities and individual variation. The most frequently reported difficulties included a lack of specialist knowledge about autism in adults among general practitioners and frontline psychologists, stereotypical conceptions of autism (limited to childhood presentations or male clinical prototypes), as well as long-standing misdiagnoses or the dismissal of reported symptoms.

In summary, it is important to recognise that an ASC diagnosis in adulthood was a landmark moment for the interviewees, often long-awaited. The confirmation of the diagnosis allowed them to better understand themselves and their needs, despite previous years of uncertainty and misdiagnosis. This reveals the need for greater awareness of ASC among mental health professionals and access to appropriate diagnostic and support services for adults with ASC. The following section explores in more detail how this new self-understanding shaped participants’ everyday functioning, relationships, and future planning.

### 3.3. Experience of the Diagnosis Process

#### 3.3.1. Long-Term Plans and Workplace Adjustments

A summary of the experiences of individuals with autism spectrum condition after receiving a diagnosis indicates the limited impact of the diagnosis itself on the life plans of the majority of the interviewees (four out of six). These individuals, despite a newly gained awareness of their own predispositions and limitations, did not significantly revise their long-term professional and educational goals. Karolina emphasises that the diagnosis has allowed her to better understand her own needs in the context of her professional work:

“In terms of the direct impact of the diagnosis on my plans, no big changes really. Somewhat indirectly maybe, because going into university I already knew that I wanted to work in a school, that I wanted to work with children but it’s thanks to the diagnosis that I know I can’t work in big groups, that group activities are not for me. I prefer to work with the child individually because I don’t get so tired. Fewer hours are better for me, I’m never likely to make it full time because I just can’t cope sensorially; it’s details like that, but the main outline hasn’t changed.”

Karolina’s story, which describes the implementation of specific organisational and communicative adjustments in the workplace following her diagnosis, represents the most explicit example of the theme of vocational adaptation. However, this theme was not unique to her account—it also appeared in the narratives of other participants, albeit to varying degrees and in different forms. Some referred to increased awareness of their own energy management, negotiating work conditions with supervisors, or avoiding sensory overload.

Milena emphasises that the diagnosis has given her confidence in her ability to find employment. Her growing self-confidence following her diagnosis is particularly compelling. She described the diagnosis as opening new possibilities and helping her understand previously confusing behaviours. This reflects a broader theme emerging in the data—self-image reconstruction—where the diagnosis seems to be a “turning point” allowing for reinterpreting one’s life history. The process of receiving a diagnosis in adulthood enabled a shift from internalised stigma or confusion toward increased self-acceptance.

#### 3.3.2. Diagnosis as One of Many Factors Leading to Change

A noteworthy theme that emerged was the significance of shared ASC identity within intimate relationships. Adam described how the diagnosis played an important role in the development of his relationship with a partner who is also on the spectrum, facilitating mutual understanding and emotional alignment. Similarly, Marta highlighted how her diagnosis helped her partner understand and accommodate her needs more effectively. These examples suggest that shared or well-understood neurodivergence in romantic relationships can serve as a source of relational strength, providing emotional safety and reducing misunderstandings rooted in neurotypical assumptions. Other participants in the study, such as Marta, indicated that changes in their lives, such as changing jobs or postponing studies, were not directly linked to the diagnosis. The same applies to Anna, who notes that although her plans became more precise, this was not the result of the diagnosis. This distinction between life changes directly resulting from the diagnosis and those emerging independently highlights the complexity of interpreting causality in retrospective self-narratives. It suggests that while diagnosis can be a turning point in self-understanding, it often operates alongside other developmental, relational, and contextual factors, complicating linear models of psychological or social change.

Significantly, the family and social relationships of the respondents remained mostly unchanged after the diagnosis, which may reflect the stability of these relationships or a reluctance to share information about the diagnosis outside of a small circle of loved ones. Only Karolina experienced an improvement in her relationship with her family, who could then better understand the reasons for her behaviour. This reluctance to disclose the diagnosis more broadly appears to be shaped by concerns about social stigma and the fear of being misunderstood or labelled. For some participants, the decision to conceal their diagnosis can be seen as a “protective strategy”, aimed at preserving existing social roles or avoiding unwelcome attention. This aligns with broader findings in autism research on the phenomenon of camouflaging, where autistic individuals modify or hide aspects of their identity to conform to neurotypical expectations. In this context, non-disclosure can be seen as a continuation of these adaptive—but potentially psychologically costly—strategies, raising important questions about social acceptance and the emotional burden of concealment.

#### 3.3.3. Recognising Needs and Accessing Support

With regard to private and professional life, most study participants have noticed some changes after their diagnosis, such as a better adaptation of work to their needs (Marta) or increased self-confidence influencing life decisions (Anna).

The diagnosis also seems to have facilitated the interviewees to seek support and understanding from groups of people with ASC and to use career counselling services for people with disabilities (Milena). Participants emphasised that, although receiving a diagnosis represented an important moment of self-identification, the lack of adequate and easily accessible forms of support (e.g., psychological consultations, peer support groups, information about rights) led to feelings of abandonment and confusion. For some, the diagnosis opened the way to seeking new sources of assistance. This theme revealed structural gaps in the support system for autistic adults. Adam notes:

“Well, as far as work is concerned, I absolutely didn’t change it; in fact it was important to me, it had a stabilising effect. It was important to me that I’ve had the same job, because it’s something secure. At that time when I was having a hard time to embrace my mental condition and a lot of things were affected by it, it was important to me that I had this steady, stable job.”

This observation suggests that diagnosis may also serve an emotional regulatory function during periods of psychological distress. For Adam, maintaining a stable job amidst his mental health challenges provided a sense of security. The diagnostic process may contribute to stabilising one’s emotional state by offering a coherent explanation for his/her experiences and being able to access relevant services.

Obtaining an ASC diagnosis in adulthood does not lead to significant changes in the life plans of the majority of the interviewees, but it contributes to a better understanding of oneself and one’s needs, which can have positive consequences in interpersonal, professional and educational aspects.

In summary, key themes emerging from the interviews include enhanced self-understanding, a selective sharing of their diagnosis, improved adaptation to life circumstances, and the use of their diagnosis as a tool for relational and professional navigation.

### 3.4. Life After Diagnosis and Evaluation of Its Effects

#### 3.4.1. Diagnosis as Transformative Event

When analysing the experiences of the study participants after receiving the diagnosis, it can be noticed that they unanimously emphasise the positive impact of the diagnosis on their self-awareness and quality of life. The diagnosis allowed the interviewees to better understand themselves, including their needs, preferences and limitations, which translates into making conscious life choices. The findings clearly indicate that a diagnosis acts as a transformative event, fostering deeper self-understanding and enabling participants to reinterpret their past and future through a more compassionate and coherent lens. In our view, this is a strong argument to take a closer look at the diagnosis process itself, not merely as a medical classifying tool but as an existentially important event. This means, e.g., that greater pressure should be put on the pre- and post-diagnosis periods and the clinical significance of the diagnosis should extend far beyond the mere fact of observing symptoms and meeting in an expert’s office. Anna cites that the diagnosis made it easier for her to choose a suitable job (“Oh yes. That’s what made it easier for me to choose, for example, a job, but apart from that, I got to know myself better in general and it’s just easier for me to live my life”), while Marta draws attention to a better understanding of her own sensory and emotional reactions:

“Yes, I mean I think I can name a lot of things, that is, it’s not that I don’t know what’s going on, but I know I’m overstimulated. I think I try to choose things that serve me well; for example, I know I hate it when there’s a lot of people and when they’re talking through a megaphone [a reference to a sporting event the interviewee’s boyfriend takes part in] so I don’t force myself to be in places like that and I just know that I don’t have to force myself to do it and I accept that, whereas before that I had a sense of obligation that made me terribly tired. I’m still very much noticing how I’m getting a focus on something, a fixation, and I stop reproaching myself that I’m dealing with such things because it is pleasurable for me. I’m constantly trying out for myself what serves me and what doesn’t, and I’m just simply better off.”

Anna’s account reflects how diagnosis can enhance personal agency by equipping individuals with the knowledge to make informed, self-supportive decisions in line with their needs and capabilities. In this context, agency refers to the individual’s capacity to act intentionally and make choices that affect their life trajectory, especially in situations previously shaped by confusion or external expectations. The process of naming and understanding one’s neurodivergence can restore a sense of authorship over one’s experiences and future direction [38]. While Marta describes classic sensory sensitivities, these may also be intertwined with attentional regulation difficulties. Attentional processes—such as difficulties in shifting focus, filtering irrelevant stimuli, or sustaining attention—can significantly modulate how sensory input is experienced. For example, heightened focus on specific sensory stimuli may amplify discomfort, while an inability to disengage from overwhelming input can exacerbate distress. A more multifactorial interpretation that includes sensory–attentional dynamics offers a broader framework for understanding these experiences.

#### 3.4.2. Diagnosis Impacts the Sense of Agency and Important Life Choices

Karolina recognises the significant role of psychotherapy in the process of getting to know oneself. In this light, psychotherapy can be seen as a catalyst that amplifies the benefits of diagnosis, facilitating the reconstruction of self-image and promoting adaptive life changes. Milena points to the diagnosis as a source of understanding the causes of certain behaviours and opening up new possibilities (“To some extent it explained the causes of certain behaviours and opened up new possibilities”).

After receiving the diagnosis, Milena created a “guide” for her loved ones to understand where some of her behaviours come from and what autism is all about.

“Yes, the kind of closest people I often spend time with have definitely started to understand me better. I’ve told them a lot, I’ve created a sort of handbook with snippets from various articles about what is due to my autism and what isn’t, and they’ve actually learnt why I behave in this way and not in that way, and they’ve started to take that into account when, for instance, planning time together.”

In several cases, the diagnosis was followed by improvements in both relationships and career-related decisions. Karolina mentioned better communication with her family, who started to understand her behaviour more clearly. Milena created a guide about autism for her close ones. When it comes to work and education, Anna said the diagnosis helped her make clearer choices about her career path. Marta noted that understanding her sensory and emotional needs made it easier to choose environments that suited her. Milena also reported feeling more confident about using support services for people with disabilities. These examples suggest a broader pattern: diagnosis helps people make better, more informed decisions in both personal and professional areas of life. Milena’s “guide” for her family is an example of a grassroots relational strategy grounded in increased self-awareness and communicative transparency. Such practices may function as relational tools that foster understanding within the close social environment. Although this theme appeared only once in the present study, it warrants further exploration in future qualitative research. From a practical perspective, investigating such strategies could inform the development of support interventions focused on family and partner relationships among autistic adults.

#### 3.4.3. Diagnosis as a Chance to Reframe One’s Past

Despite the difficulties of living with ASC, the interviewees point to many positive aspects of the diagnosis. A recurring and particularly powerful outcome was the development of greater self-acceptance, which several participants described as transformative. The diagnosis allowed them to better understand themselves, reframe past difficulties, and stop blaming themselves for behaviours they previously saw as personal failings. It also opened up access to specialist support and improved communication with loved ones. Daria stated:

“You know more about yourself, you also stop blaming yourself for some things, you can also get some facilities when, for example, like me you are registered as autistic at university. For me as a parent, it’s also important that I’m able to help my child avoid those problems that I struggled with if she turned out to be autistic too.”

Daria’s narrative, in which she reflects on her role as a mother, represents an important perspective that highlights the impact of diagnosis not only on individual self-understanding but also on caregiving and relational roles. Receiving an ASC diagnosis enabled her to develop a new awareness of both her own boundaries and her child’s needs, leading to a more intentional and empathetic approach to everyday family interactions. This theme illustrates that a late diagnosis can bring about change not only in personal functioning but also in relationships, rooted in care, responsibility, and mutual understanding. Diagnosis is seen as a tool to better adapt life to one’s needs and limitations. In conclusion, a diagnosis of ASC in adulthood has significant benefits in terms of self-awareness and quality of life. Improved relationships with loved ones, a better understanding of one’s own needs and limitations, and access to support and specialist services are key to building a fulfilling life with ASC.

Overall, diagnosis appears to have the strongest reported impact in three domains: increased self-knowledge, enhanced interpersonal understanding, and improved life decision-making processes.

## 4. Discussion

The findings of this study point to the need for a more nuanced understanding of adult autism diagnosis—one that goes beyond access and procedural efficiency to include how the diagnosis is interpreted and integrated by the individual. Rather than viewing diagnosis as a purely clinical endpoint, the participants’ narratives reveal it as a complex social and psychological process—one that involves the negotiation of identity, the re-evaluation of relationships, and the navigation of societal expectations. The diverse experiences captured in this study suggest that while diagnosis can be empowering and clarifying, it can also produce tension, especially when professional frameworks fail to align with lived experience. These insights call for a critical rethinking of diagnostic practices and the epistemic roles of both clinicians and autistic individuals in shaping what diagnosis ultimately means. Adults with ASC often face difficulties in accessing an adequate diagnosis, which may be due to professionals’ lack of knowledge about the manifestations of ASC in adulthood and stereotypical perceptions of the condition [39]. These knowledge gaps could be addressed through training in neurodiversity-informed care, increased collaboration with autistic self-advocates, and the incorporation of first-person narratives into professional education. The literature also suggests several strategies to deal with these difficulties on systemic and personal levels [40,41].

Emphasising the positive impact of the diagnosis on the self-awareness and quality of life of the study participants points to the importance of early diagnosis and the adaptation of support. The adaptive value of the diagnosis was primarily expressed in three areas: (1) the reorganisation of daily functioning, (2) increased self-acceptance and reduced internal tension, and (3) the development of more realistic expectations toward oneself. An ASC diagnosis can facilitate an understanding of one’s own limitations and predispositions, which in turn contributes to a better adaptation of the work environment, education, and private life to the needs of the individual with ASC. In the context of interpersonal relationships, diagnosis often leads to improved understanding and acceptance from loved ones, which is important for the quality of life of individuals with ASC [42].

It is also important to draw attention to those participants who described negative experiences during the diagnostic process, such as a lack of clear feedback about their test results or unprofessional comments from clinicians. These accounts point to a broader issue increasingly seen in adult autism diagnosis: the tension between self-identification—often shaped by online tools and growing familiarity with neurodivergent perspectives—and the outcomes of formal clinical assessment. Some individuals arrive at the diagnostic process already convinced that they are autistic. When the evaluation does not confirm this belief, or when the process feels impersonal or dismissive, it can lead to frustration, disappointment, and a lack of trust in professionals. In some cases, this mismatch encourages people to seek out additional opinions or repeat assessments in search of validation—a phenomenon sometimes referred to as “diagnostic shopping.” These dynamics suggest the need for a more open and dialogical approach to diagnosis, one that respects lived experience and creates space for genuine engagement between clinician and client. The diagnosis also had a clear impact on how participants saw themselves and related to others. Many of them described feeling more self-aware and more accepting of who they are after receiving the diagnosis. This, in turn, helped them manage everyday challenges more effectively and build more fulfilling social relationships. Seen through the lens of grassroots epistemology, this process reflects something more than just receiving a medical label—it shows how people actively use the diagnosis to make sense of their past and present. Instead of accepting external definitions passively, participants reinterpreted their experiences in a way that felt authentic and personally meaningful. In this sense, the diagnosis became a turning point: a moment that helped them reclaim ownership over how they understand their own minds and lives. It is worth noting, however, that a diagnosis does not always involve significant changes in life plans. Rather than being neutral, the absence of these changes can reflect a dynamic tension between newfound self-awareness and pre-existing social roles or expectations that resist change.

Ultimately, an ASC diagnosis in adulthood is an important step towards a better understanding of oneself and building a life that complies with individual needs and limitations. In his recent study summarising the experience of an autism diagnosis, Kiehl [11] noted that:

“While the diagnostic process was confusing and disappointing for many, it often led to a sense of relief and clarity regarding past experiences. It created opportunities to connect with other autistic individuals and to access services […]”

As in the case of Kiehl’s study, while most participants perceived the diagnosis as a turning point, the scope and intensity of its impact varied considerably. For some, it marked a profound moment of identity reinterpretation and served as a catalyst for consciously adapting everyday life to better fit their needs—whether in the realm of work, relationships, or rest. Others described the diagnosis as having a more symbolic function: it offered relief and a sense of explanation for past difficulties but did not lead to major changes in daily functioning. This variation was acknowledged in the analysis, illustrating that diagnosis can serve different roles—ranging from transformative to stabilising—depending on the individual’s biographical context.

In our case, all study participants unanimously acknowledged that if they were to compare their lives before and after the diagnosis, it was definitely the one after that they found more satisfying: “Of course, after the diagnosis. I always knew I was different, I thought there was something wrong with me, and the diagnosis made me realise that nothing that happened before was my fault; it’s just the way I am and I accepted that,” says Anna. Her statement, in which she emphasises that “being herself” after the diagnosis does not signify change but rather the possibility of living more in alignment with her identity, offers a particularly powerful testament to the transformative potential of a late diagnosis. Her voice highlights that diagnosis may not so much introduce something new as restore narrative continuity to one’s personal history and strengthen the sense of authenticity.

Comparing her life before and after the diagnosis, Marta declares:

“In all seriousness, it wasn’t bad before the diagnosis until that point when it started to get bad and I would generally say that it got totally bad after the diagnosis, but that’s only because I feel I’m getting back to that good state before the diagnosis.”

Marta’s story highlights the emotional ambivalence of a late diagnosis—combining relief and self-understanding with regret over missed support and lost time. Similar feelings appeared in other accounts, reflecting how diagnosis can prompt both clarity and reflection on past unmet needs. This ambivalence may be seen as part of the emotional cost of late diagnosis, shaping identity and adaptation.

Adam talks about how, owing to the diagnosis, he has realised that he is not a person who is “devoid of emotion” but simply has difficulty reading his emotions, and if he had discovered this earlier, it would surely have been easier for him:

“Well I don’t know, because that’s how I thought I was before, that I was just mindful, acting on logic, devoid of emotion, and then it turned out that I wasn’t because I got caught up in those emotions, I just didn’t know how to read them, so until a certain point I didn’t see it as a problem, and I think if I had known that earlier it would have been easier for me.”

Adam’s statement represents a significant example of enhanced emotional insight as part of the self-discovery process. This aspect of the experience can be interpreted as a manifestation of broader identity reconfiguration—not only through the acquisition of a new language for self-description but also through a restructuring of one’s relationship with inner emotional states. Here, the diagnosis serves as a point of departure for constructing a more integrated self-concept, in which emotional functioning—previously misunderstood or suppressed—gains recognition and meaning.

Karolina claims that the diagnosis has made a huge difference to her and has significantly improved the quality of her life.

“When I look at my life about five years ago and now, it’s a completely different life. Now I know how to schedule my day so that it’s right for me, I know that if I have a lot of work one day then the next day I need to rest. In the same way when I was going to work and there was a schedule I knew I couldn’t go to work at 7.30 am every day because I would just be inefficient. I’ve made a lot of progress with psychotherapy and it’s made a big difference to my life, so it’s hard for me to judge objectively, but overall it’s definitely been a lot easier for me after the diagnosis.”

The improvements in daily organisation reported by Karolina—such as more effective task planning, careful energy management, and clearer boundary-setting—can be interpreted not only as a direct outcome of receiving a diagnosis but also as a result of the psychotherapeutic process that the diagnosis either initiated or reinforced. In her narrative, Karolina refers to engaging in psychotherapy, which suggests that the development of new organisational strategies took place within a supportive and reflective relational context. Thus, improvements in executive functioning and self-regulation may be understood as the product of a synergy between diagnostic insight and therapeutic work. The findings also highlight the importance of the availability of specialist diagnostic and support services [43], as well as the need for public education about ASC to counter stereotypes and promote the social inclusion of people with ASC [44,45]. It is important to emphasise that adults with a late ASC diagnosis still face limited access to specialised diagnostic and therapeutic services. There is a shortage of centres specifically dedicated to diagnosing autism in adulthood, as well as a lack of structured programmes supporting neurodivergent individuals outside the disability certification system. At the policy level, it is crucial to recognise a late diagnosis not merely as a formal label but as a legitimate point of entry into support systems with practical follow-up implications. Marta mentions imposter syndrome and the fear of stigma:

“I have imposter syndrome. It’s just that when I see someone ‘more autistic than me’ it makes me think that I made up this diagnosis, that they only gave it to me because I paid for it. Such silly thoughts just come to me, and the other is a fear, though slight, of stigmatisation.”

Those fears, explicitly expressed in Marta’s account, constitute a significant interpretive thread. Concern about disclosing the diagnosis and the potential reactions of others—including stigma, marginalisation, or misunderstanding—also emerged implicitly in other narratives, for example in the context of selectively informing colleagues or avoiding formal inclusion of the diagnosis in professional documentation. This fear did not pertain solely to the label of “autism” itself but also to the broader social consequences of deviating from normative functioning, particularly within professional and family environments. In this context, camouflaging emerged as a common strategy to navigate social spaces while concealing aspects of autistic identity—often at a psychological cost. As a cross-cutting theme, stigma forms part of the analysis of the identity-related and adaptive costs of a late diagnosis, revealing the tension between the need for recognition and the need to protect oneself from social judgment.

The findings of this study suggest that future research should focus more closely on (1) the psychosocial consequences of a late ASC diagnosis in the context of employment, family life, and intimate relationships; (2) adaptive and communicative strategies developed post-diagnosis, both individually and in social interactions; and (3) experiences of stigma and emotional ambivalence as enduring components of the process of integrating an autistic identity in adulthood. From a practical perspective, implementation research is recommended on post-diagnostic support models that combine elements of psychoeducation, relational intervention, and identity-based support. This includes the development of tools that facilitate mutual understanding in close relationships—such as the personal “guide” created by Milena for her family—which may help bridge communication gaps and reduce misunderstanding. Such an approach would better address the diverse needs of autistic individuals diagnosed after the age of 18.

## 5. Conclusions

The reported study implies the following conclusions:

- Grassroots epistemology plays a crucial role in diagnosing and understanding the experiences of individuals on the autism spectrum. This approach values firsthand perspectives and insights from those directly affected by autism, promoting a deeper and more nuanced comprehension of their unique experiences. By emphasising the voices of autistic individuals themselves, grassroots epistemology challenges traditional, top-down diagnostic frameworks that often overlook the personal narratives and subjective experiences crucial for a holistic understanding.
- There is an urgent need to increase the availability and improve the specialisation of diagnostic services for adults with ASC. Action needs to be taken to raise awareness among mental health professionals of the specificity of ASC in adulthood and to break down stereotypical perceptions of this condition.
- A diagnosis of ASC has a positive impact on the self-awareness and quality of life of individuals with ASC, enabling them to better understand their own limitations and predispositions. This points to the importance of early diagnosis and appropriate support.
- Negative experiences of the diagnostic process, such as the lack of detailed discussion of test results, highlight the need to improve diagnostic and therapeutic practices to be more tailored to the specific needs of individuals with ASC.
- An ASC diagnosis can lead to a change in self-perception and relationships, contributing to an increase in self-acceptance and an improvement in the quality of interpersonal relationships.

Suggested directions for further research are as follows:

- Further research should be focused on a detailed analysis of the impact of diagnosis on various aspects of the lives of people with ASC, including their professional, educational, and social relationships.
- Research is needed to improve diagnostic and therapeutic methods so that they are more sensitive and tailored to the needs of individuals with ASC.
- Long-term research should be conducted to assess the long-term impact of an ASC diagnosis on the quality of life, self-awareness, and social functioning of people with this condition.

In summary, an ASC diagnosis in adulthood paves the way to a better understanding of oneself and building a life tailored to individual needs and limitations. Further research and development of services are crucial to improve the quality of life of individuals with ASC and to realise their full potential.

## 6. Limitations

This study is based on a qualitative methodology, which entails certain limitations. The small sample size, while often seen as a flaw in quantitative research, is an intentional feature here. It allows for in-depth access to participants’ lived experiences—something not achievable in large-scale studies. This approach is grounded in grassroots epistemology, which values first-person perspectives and centres the knowledge of marginalised groups, such as autistic individuals [46].

In conducting this study, we prioritised the perspectives of autistic individuals by using semi-structured interviews. This approach reflects a participatory ethos, treating autistic people not just as subjects of research but as co-constructors of knowledge. At the same time, this method has its limitations. Semi-structured interviews depend on participants’ willingness and ability to articulate their experiences verbally, which may privilege certain communicative styles over others. Some nuances of lived experience might remain unspoken or difficult to express, particularly in a context where emotional, sensory, or cognitive factors affect communication. The interpretive nature of the method also introduces a degree of researcher mediation, which, although reflexively managed, cannot be entirely eliminated.

The absence of quantitative measures before and after diagnosis reflects the study’s aim: not to assess outcomes numerically but to explore how people make sense of their diagnosis in their own terms. While this introduces subjectivity, such subjectivity is essential in capturing the personal and contextual nature of meaning-making processes.

Nonetheless, these strengths also present limitations. The findings should not be interpreted as representative of all autistic people, and the subjective narratives analysed here cannot substitute for longitudinal or large-scale studies using mixed methods. Future research could integrate qualitative insights with quantitative tools to triangulate data and increase robustness, particularly concerning the long-term psychosocial effects of diagnosis.

## Figures and Tables

**Table 1:** Participants overview.

Name *	Age Current (Diagnosis)	Gender	Family Status	Education	Occupation	Residence
Anna	29 (27)	Female	Unmarried, informal relationship, no children	Incomplete university	Bank	Provincial city
Marta	22 (21)	Female	Unmarried, no children	Secondary	IT company	Large city
Adam	31 (31)	Male	Unmarried, informal relationship, no children	University	IT specialist (remote)	Large city
Daria	36 (34)	Female	Married, one child (daughter, aged 3)	University	Publishing house	Large city abroad
Karolina	25 (21)	Female	Unmarried, informal relationship, no children	University	School	Large provincial city
Milena	33 (32)	Transgender woman (pre-transition)	Unmarried, no children	University	Unemployed	Medium-sized city

* Names were changed due to anonymity reasons.

## Data Availability

The raw data is unavailable due to privacy restrictions.

## Abbreviations

The following abbreviations are used in this manuscript:

ASC	Autism spectrum condition
DSM	Diagnostic and Statistical Manual of Mental Disorders
ICD	International Classification of Diseases
